# Timing of rehabilitation on length of stay and cost in patients with hip or knee joint arthroplasty: A systematic review with meta-analysis

**DOI:** 10.1371/journal.pone.0178295

**Published:** 2017-06-02

**Authors:** Michael Masaracchio, William J. Hanney, Xinliang Liu, Morey Kolber, Kaitlin Kirker

**Affiliations:** 1 Department of Physical Therapy, Long Island University, Brooklyn, New York, United States of America; 2 Department of Health Professions, University of Central Florida, Orlando, Florida, United States of America; 3 Department of Health Management and Informatics, University of Central Florida, Orlando, Florida, United States of America; 4 Department of Physical Therapy, Nova Southeastern University, Fort Lauderdale, Florida, United States of America; Harvard Medical School/BIDMC, UNITED STATES

## Abstract

**Objective:**

To investigate the role of early initiation of rehabilitation on length of stay (LOS) and cost following total hip arthroplasty, total knee arthroplasty, or unicompartmental knee arthroplasty.

**Data sources:**

Electronic databases PubMed, CINAHL, Pedro, Embase, AMED, and the Cochrane Library were searched in July 2016. Five additional trials were identified through reference list scanning.

**Study selection:**

Eligible studies were published in English language peer-reviewed journals; included participants that had undergone total hip arthroplasty, total knee arthroplasty, or unicompartmental knee arthroplasty reported clearly defined timing of rehabilitation onset for at least two groups; and reported at least one measure of LOS or cost. Inclusion criteria were applied by 2 independent authors, with disagreements being determined by a third author. Searching identified 1,029 potential articles, of which 17 studies with 26,614 participants met the inclusion criteria.

**Data extraction:**

Data was extracted independently by 2 authors, with disagreements being determined by a third author. Methodological quality of each study was evaluated independently by 2 authors using the Downs and Black checklist. Pooled analyses were analyzed using a random-effects model with inverse variance methods to calculate standardized mean differences (SMD) and 95% confidence intervals for LOS.

**Data synthesis:**

When compared with standard care, early initiation of physical therapy demonstrated a decrease in length of stay for the 4 randomized clinical trials (SMD = -1.90; 95% CI -2.76 to -1.05; I^2^ = 93%) and for the quasi-experimental and 5 prospective studies (SMD = -1.47; 95% CI -1.85 to -1.10; I^2^ = 88%).

**Conclusion:**

Early initiation of rehabilitation following total hip arthroplasty, total knee arthroplasty, or unicompartmental knee arthroplasty is associated with a shorter LOS, a lower overall cost, with no evidence of an increased number of adverse reactions. Additional high quality studies with standardized methodology are needed to further examine the impact of early initiation of physical therapy among patients with joint replacement procedures.

## Introduction

With the advancement of medicine, life expectancy of the United States (US) population continues to rise. Research suggests between the year 2000 and 2050 the number of individuals living over the age of 65 will increase by 135% [1]. By the year 2050, there will be more than 69 million Americans over the age of 65, with 19 million older than 85 years of age [2]. As the population continues to age, one of the major concerns in society will be the costs associated with treating chronic musculoskeletal conditions [3]. An analysis based on a national representative survey finds that 25% of physical therapy episodes are among Medicare beneficiaries [4]. The World Health Organization has identified four chronic musculoskeletal conditions that will continue to become more prevalent as the population ages. Two of these conditions include osteoarthritis (OA) and rheumatoid arthritis (RA), both of which affect millions of individuals worldwide [5–7].

OA affects approximately 27 million adults in the US alone [8]. It is characterized by progressive articular cartilage breakdown that often leads to pain and loss of function [9]. OA costs an average of $2,600 a year per patient, with combined direct and indirect annual costs averaging $5,700 for each individual [8, 10, 11]. In addition, over 80% of those with OA present with some degree of movement limitation, and 40% of these individuals rate their quality of life as fair or poor [12]. RA is a systemic, autoimmune disease that affects approximately 1.5 million adults in the US [8]. It is characterized by destruction of the cartilage and synovial lining of multiple joints in the musculoskeletal system, which often leads to pain and functional loss [13]. RA costs an average of $2,085 a year per patient, with combined direct and indirect costs averaging $3,200 per patient each year in the US [14–17]. In addition, individuals with RA are 40% more likely to report their health as fair or poor, and 30% more likely to require assistance with daily personal care [18].

When conservative management of RA and OA has failed and the overall quality of life continues to decline for an individual, total hip and knee arthroplasty are the surgical treatments of choice to alleviate joint destruction, decrease pain, and improve quality of life [19–22]. It is predicted that by the year 2030, there will be approximately 3.5 million primary total knee arthroplasties (TKA) and almost 600,000 primary total hip arthroplasties (THA) performed each year in the US [9]. The estimated US hospital expenditures of THA and TKA surgery in 2009 were 13.7 billion and 28.5 billion dollars, respectively [23]. Unless improvements with conservative care dramatically change, it is reasonable to assume that these trends in costs will continue to rise as the population ages and a greater number of individuals elect to have these surgical procedures.

Physical therapy is an integral component in the management of musculoskeletal conditions. One study reported a direct connection between duration of physical therapy and the improvement in functional status of patients hospitalized for lower extremity orthopedic issues [24]. In addition, early initiation of physical therapy has been found to improve health outcomes among patients with cerebrovascular accident and low back pain [25–27]. A recent systematic review published by Ojha *et al* [28] favorably reported the cost-effectiveness of early physical therapy for a variety of musculoskeletal conditions without compromising patient outcomes.

Physical therapy has been shown to reduce swelling, increase range of motion, improve strength, and return individuals to a higher level of function following THA and TKA [19–22]. Inpatient physical therapy services are commonly utilized following THA or TKA and are often considered an important part of post-operative management. Over the past decade, research has focused on early post-operative physical therapy with these patients and its impact on functional outcomes, costs, length of stay (LOS), and adverse reactions in the inpatient setting [29–40].

A recent systematic review [41], which included five randomized clinical trials (RCT) assessed the role of early mobilization after hip or knee joint arthroplasty on LOS in the acute care setting. Clinical homogenous data were analyzed using meta-analysis and the results demonstrated a reduced LOS of approximately 1.8 days without increasing the risk of negative outcomes. While the Guerra *et al* [41] systematic review reported a positive result on LOS in the acute care setting, several limitations exist. First, only RCTs were included, which led to an overall small sample size (622 participants). Secondly, it only examined the impact of early mobilization on LOS and did not discuss associated cost savings.

Further research is needed to investigate the overall costs of care, in addition to the clinical effectiveness demonstrated. Considering that there will be a significant increase in THA and TKA over the next 15 years, a more comprehensive systematic review, which includes various study designs that analyze both LOS and cost effectiveness is warranted to synthesize evidence regarding the safety and effectiveness, as well as economic impact of early physical therapy initiation after joint replacement procedures.

Therefore, the purpose of this manuscript was to conduct a systematic review of the literature analyzing the role that early initiation of physical therapy has on LOS and cost following THA, TKA, or uni-compartmental knee arthroplasty (UKA). The review will provide a summary of existing literature on acute management strategies following hip or knee arthroplasty, and subsequently identify potential gaps in the literature that may lead to future inquires in this line of research.

## Methods

This article is a systematic review with meta-analysis that followed the PRISMA [42] guidelines (S1 Table. PRISMA 2009 Checklist.). This manuscript reviewed management of post-operative joint replacement in the acute care setting and the influence that early physical therapy initiation had on LOS and costs.

### Identification and selection of trials

An electronic search was conducted in July 2016 using the databases PubMed, CINAHL (EBOSCO Host), Pedro, and AMED (Ovid), Embase, and the Cochrane Library for all pertinent articles relevant to this systematic review. Key words were used independently and in combination including early rehabilitation, costs, early mobilization, early rehabilitation, hip arthroplasty, immediate physical therapy, joint arthroplasty, knee arthroplasty, length of stay, and outcomes. Specific search strategies are outlined in Table 1. The goal behind the search strategy was to identify all potential articles that discussed THA, TKA, or UKA and the role that early rehabilitation had on LOS and/or costs following surgery. After the computerized search was completed, reference lists of all selected articles were searched by hand to identify any other related articles pertaining to this research study. One author, (MM), examined all titles and abstracts to determine initial study eligibility. Full text articles were then revaluated for specific inclusion criterion. A second author, (KK), independently reviewed all full text articles for eligibility. A third author, (WJH), determined final eligibility should a discrepancy exist between the initial authors.

**Table 1:** Database search strategy.

Database	Search Strategy	Results
PubMed	• (early rehabilitation) AND (knee arthroplasty) AND ("outcomes”)	124
	• (early rehabilitation) AND (hip arthroplasty) AND (outcomes)	103
	• (early mobilization) AND (knee arthroplasty OR hip arthroplasty) AND (outcomes AND costs)	2
	• (“immediate physical therapy”) AND (“joint arthroplasty”)	0
	• (“accelerated rehabilitation”) AND (“joint arthroplasty”)	41
	• ((accelerated rehabilitation) AND joint arthroplasty) AND length of stay	0
CINAHL (EBSCO Host)	• early rehabilitation (select a field (optional) AND knee arthroplasty (select a field (optional) AND outcomes (select a field (optional)	8
	• early rehabilitation (select a field (optional) AND hip arthroplasty (select a field (optional) AND outcomes(select a field (optional)	3
	• early mobilization (select a field (optional) AND knee arthroplasty (select a field (optional) AND costs (select a field (optional)	0
	• early mobilization (select a field (optional) AND hip arthroplasty (select a field (optional) AND costs (select a field (optional)	0
	• immediate physical therapy (select a field (optional) AND joint arthroplasty(select a field (optional)	0
	• accelerated rehabilitation (select a field (optional) AND joint arthroplasty (select a field (optional)	0
AMED (Ovid)	• early rehabilitation AND joint arthroplasty AND	52
	• accelerated rehabilitation AND joint arthroplasty AND length of stay	13
	• accelerated rehabilitation AND joint arthroplasty AND costs	15
	• early mobilization AND (knee arthroplasty OR hip arthroplasty) AND outcomes	351
PEDro	• Simple search: early rehabilitation, knee arthroplasty, outcomes	1
	• Simple search: early rehabilitation, hip arthroplasty, outcomes	1
	• Simple search: early mobilization, knee arthroplasty, hip arthroplasty, outcomes, costs	0
	• Simple search: immediate physical therapy, joint arthroplasty	0
	• Simple search: accelerated rehabilitation, joint arthroplasty	2
	• Simple search: accelerated rehabilitation, joint arthroplasty, length of stay	0
Embase	• (early AND (‘rehabilitation’ OR rehabilitation)) AND (‘knee” OR knee AND (‘arthroplasty’ OR arthroplasty)) AND (outcomes)	74
	• (early AND (‘rehabilitation’ OR rehabilitation)) AND (‘hip’ OR hip AND (‘arthroplasty’ OR arthroplasty)) AND (outcomes)	71
	• (early AND (‘mobilization’ OR mobilization)) AND ((‘knee’ OR knee AND (‘arthroplasty’ OR arthroplasty) OR (‘hip’ OR hip AND (‘arthroplasty’ OR arthroplasty)) AND outcomes AND costs	5
	• ((immediate AND physical AND (‘therapy’ OR therapy)) OR (immediate AND (‘physiotherapy’ OR physiotherapy))) AND (‘joint’ OR joint AND (‘arthroplasty’ OR arthroplasty))	30
	• (accelerated AND (‘rehabilitation’ OR rehabilitation)) AND (‘joint’ OR joint AND (‘arthroplasty’ OR arthroplasty))	18
Cochrane	• early rehabilitation AND joint arthroplasty	1
	• early mobilization AND joint arthroplasty	1
	• accelerated rehabilitation AND joint arthroplasty	9
	• accelerated rehabilitation AND joint arthroplasty AND length of stay	0
	• accelerated rehabilitation AND length of stay	38
	• accelerated rehabilitation AND costs	9
	• accelerated rehabilitation AND arthroplasty AND outcomes	5

#### Inclusion criteria

To be included in this systematic review, an article needed to meet the following inclusion criteria: (1) published in a peer reviewed journal; (2) published in the English language; (3) at least 2 groups in which the definition of timing of rehabilitation was clearly stated; (4) at least one measure of LOS or cost; (5) patients undergoing THA, TKA, or UKA.

#### Exclusion criteria

Case studies and case series were excluded. Also, any potential disagreements were resolved through consensus between two authors, (MM and KK), and if consensus could not be agreed upon, a third author, (WJH), was consulted for a final opinion.

#### Outcomes

The primary outcomes for this systematic review were LOS and costs following joint arthroplasty surgery of the hip and knee. For the purpose of this review LOS was defined as the number of days spent in the hospital from surgery to discharge from the acute care setting. Costs, on the other hand, were defined differently among the included studies. Chen *et al* [30] defined total medical expenses as rehabilitation expenses related to rehabilitation services in the index visit, as well as outpatient and inpatient expenses related to prosthetic infection and deep vein thrombosis within one year after discharge. Larsen *et al* [34] defined total medical expenses as costs associated with the information day, hospital stay, care in the hospital, rehabilitation in the hospital, patient needs, primary care in the follow-up period, and hospital re-admission in the follow-up period of one year after discharge. Reilly *et al* [43] defined total average costs as the combination of fixed costs (surgery, anesthesia, prosthesis, etc), average hospital stay cost, additional outpatient appointments, and cost of specialist registrar time over a six month period. Lastly, Pua and Ong [37] solely assessed hospitalization costs at discharge, which included room and ward charges, professional fees, laboratory investigations, pharmaceutical supplies, implant, and rehabilitation services.

### Assessment of characteristics of trials

#### Quality assessment of trials and risk of bias

A modified version of the Downs and Black checklist [44] was chosen to evaluate the methodological quality of the articles in this systematic review because it provided the ability to use one standardized scale for both randomized and non-randomized controlled trials. For this study, item 27 was adjusted to score power using a 3-option scale versus a 6-option scale, changing the total score of the Downs and Black checklist from 32 to 28 points (S2 Table. Modified Downs and Black Checklist.). The Downs and Black checklist assesses four different categories that include reporting, external validity, internal validity/bias, and internal validity/confounding. The Downs and Black checklist has demonstrated good inter-rater reliability (r = 0.75) and test-retest reliability (r = 0.88) [44]. Each article was scored by two independent authors, (MM and KK). The results of these scoring tests were blinded for each of the evaluators, and a third author, (WJH), evaluated inconsistent scores.

#### Data extraction

Data extraction was performed by one author, (KK), and all authors were consulted with any issues or questions that were encountered during the process. If discrepancies existed, final decisions were reached via consensus of all authors. Extracted data included: study design, country of origin, participant demographics, outcome measures, interventions, and results.

#### Data analysis

Data analysis was conducted using Revman 5.3. Two meta-analyses were performed for the continuous outcome variable LOS, using a random-effects model with inverse variance methods to calculate a standard mean difference and 95% confidence intervals (CI). One meta-analysis was conducted for all randomized clinical trials [32, 36, 43, 45] and another was conducted for all prospective and quasi-experimental studies [29, 33, 35, 40, 46, 47]. Previous research [48] has provided a scale for interpreting the strength of the standard mean difference: 0.2 indicates a small effect, 0.5 indicates a medium effect, and 0.8 indicates a large effect. Statistical heterogeneity was calculated using the I^2^ statistic, with values of more than 50% indicating considerable levels of heterogeneity [49].

## Results

### Study selection

**Fig 1:**
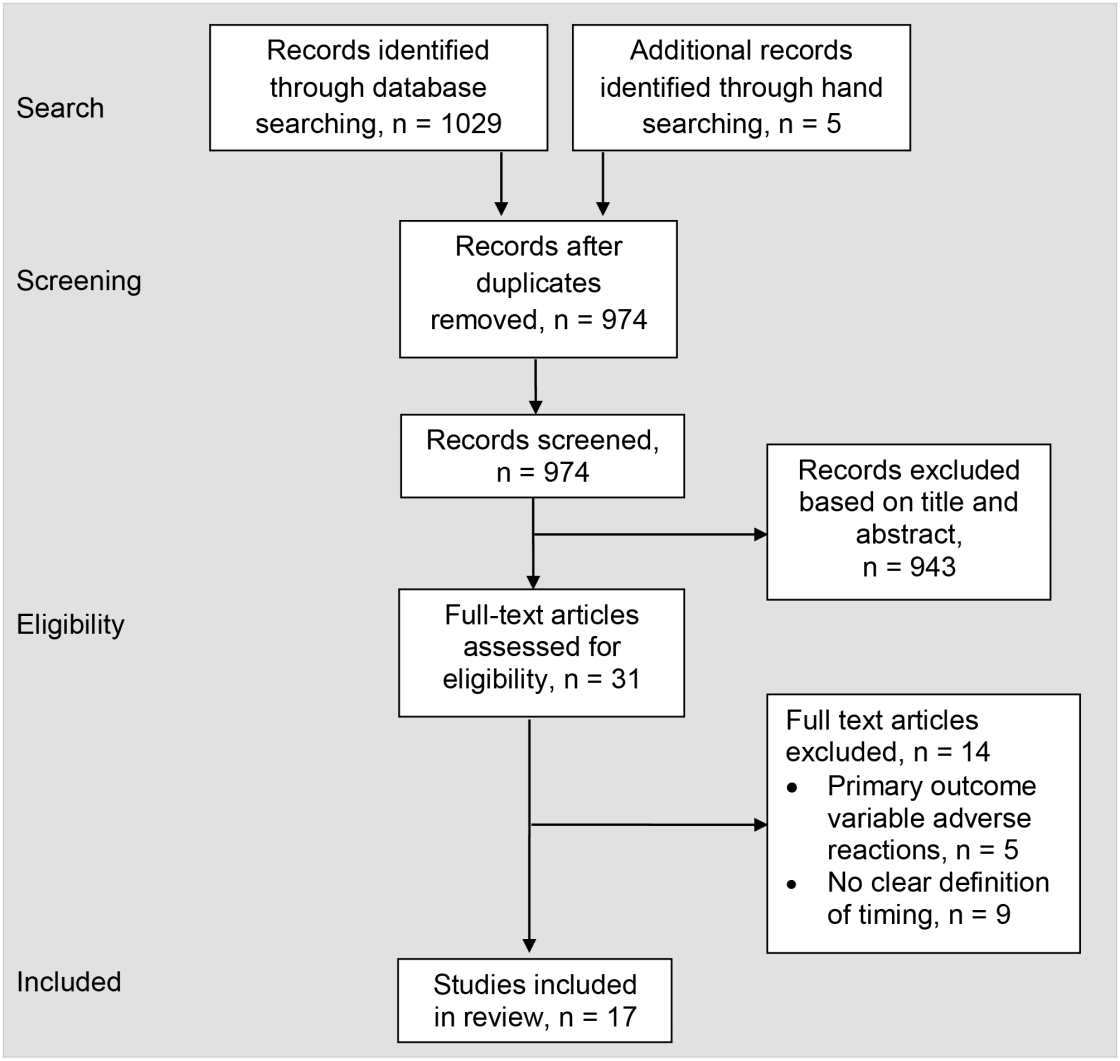
PRISMA flow diagram.

The search strategy identified 1029 studies, and five additional studies were located through manual searching. After duplicates were removed, 974 remained to be screened based on title and abstract. Thirty-one full-text articles were assessed and 17 were included for review after screening to determine eligibility (Fig 1). The 17 studies included patients who had undergone THA, TKA, and UKA and implemented a variety of inpatient rehabilitation paradigms.

### Characteristics of included trials

A total of 26,614 patients undergoing either THA, TKA, or UKA were included across all 17 studies and received either early rehabilitation or standard rehabilitation. For the purposes of this systematic review, early and standard rehabilitation are relatively defined for each individual study. Therefore, across studies, these operational definitions may overlap in regards to timeframe.

#### Quality

Seventeen studies were individually scored for their methodological quality, which are presented in Table 2. The average score across 17 studies was 21/28 on the modified Downs and Black checklist, with a range of 16–26. Overall, reduced quality across studies can be attributed to inadequate reporting of blinding, loss to follow up, randomization, and adjustment for confounding variables in the analyses. Of the 17 studies included, 4 were randomized clinical trials, [32, 36, 43, 45] 1 was a quasi-experimental study, [33] 1 was a cost-effectiveness study, [34] based on the Larsen *et al* 2008 randomized clinical trial, [36] 6 were prospective cohort studies, [29, 35, 39, 40, 46, 47] and 5 were retrospective cohort studies [30, 31, 37, 38, 50].

**Table 2:** Downs and Black methodological quality.

	Reporting										External Validity			Internal Validity-Bias-							Internal Validity-Confounding-							
Study	1	2	3	4	5	6	7	8	9	10	11	12	13	14	15	16	17	18	19	20	21	22	23	24	25	26	27	D&B Score
Chen A *et al* 2012 [6]	Y	Y	Y	Y	Y	Y	Y	N	N	Y	Y	N	Y	U	U	Y	Y	Y	Y	Y	Y	Y	N	N	N	N	Y	18/28
Chen H *et al* 2012 [7]	Y	Y	Y	Y	Y	Y	Y	Y	Y	Y	Y	Y	Y	Y	U	Y	Y	Y	Y	Y	Y	Y	N	N	Y	Y	U	24/28
den Hertog *et al* 2012 [44]	Y	Y	Y	Y	Y	Y	Y	Y	Y	Y	Y	Y	N	Y	U	Y	Y	Y	Y	Y	Y	Y	Y	Y	Y	Y	Y	26/28
Gulotta *et al* 2011 [46]	Y	Y	Y	Y	Y	Y	Y	Y	N	Y	Y	Y	Y	U	U	Y	Y	Y	Y	Y	Y	Y	N	N	Y	U	U	21/28
Isaac *et al* 2005 [15]	Y	Y	Y	Y	N	Y	Y	Y	N	Y	Y	U	Y	Y	U	Y	Y	Y	Y	Y	Y	Y	N	N	N	U	U	18/28
Juliano *et al* 2011 [17]	Y	Y	Y	Y	Y	Y	Y	N	N	Y	Y	N	Y	U	U	Y	Y	Y	Y	Y	Y	Y	N	N	N	N	Y	19/28
Labraca *et al* 2011 [20]	Y	Y	Y	Y	Y	Y	Y	N	Y	Y	Y	Y	Y	U	Y	Y	Y	Y	Y	Y	Y	Y	Y	N	N	Y	U	23/28
Larsen *et al* 2008 [21]	Y	Y	Y	Y	Y	Y	Y	N	Y	Y	Y	Y	Y	Y	U	Y	Y	Y	Y	Y	Y	N	N	N	Y	Y	Y	23/28
Larsen *et al* 2009 [22]	Y	Y	Y	Y	P	Y	Y	Y	Y	Y	Y	Y	N	Y	Y	Y	Y	Y	Y	Y	Y	Y	N	N	N	Y	U	22/28
Larsen *et al* 2008 [23]	Y	Y	Y	Y	Y	Y	Y	Y	Y	Y	Y	Y	N	Y	Y	Y	Y	Y	Y	Y	Y	Y	N	N	Y	Y	Y	24/28
Larsen *et al* 2008 [24]	Y	Y	Y	Y	P	Y	Y	Y	Y	Y	Y	Y	N	Y	U	Y	Y	Y	Y	Y	Y	N	Y	Y	Y	Y	Y	24/28
Pua & Ong 2014 [35]	Y	Y	Y	Y	Y	Y	Y	N	N	Y	Y	Y	Y	U	U	Y	Y	Y	Y	Y	Y	Y	N	N	Y	N	U	19/28
Raphael *et al* 2011 [50]	Y	Y	Y	Y	Y	Y	Y	Y	N	N	Y	N	Y	U	N	Y	Y	Y	Y	Y	Y	Y	N	N	Y	U	U	19/28
Reilly *et al* 2005 [45]	Y	Y	Y	Y	P	Y	Y	Y	Y	Y	Y	Y	Y	Y	U	Y	Y	Y	Y	Y	Y	U	Y	Y	N	Y	Y	24/28
Robbins *et al* 2014 [36]	Y	Y	Y	Y	N	Y	Y	Y	N	N	Y	N	Y	U	U	Y	Y	Y	Y	Y	Y	Y	N	N	N	N	U	16/28
Tayrose *et al* 2013 [40]	Y	Y	Y	Y	Y	Y	Y	N	N	Y	Y	N	Y	U	U	Y	Y	Y	Y	Y	Y	Y	N	N	N	N	U	19/28
Wellman *et al* 2011 [42]	Y	Y	Y	Y	N	Y	Y	Y	N	N	Y	Y	Y	U	U	Y	Y	U	Y	Y	Y	Y	N	N	N	N	U	16/28

Criteria based on Downs and Black check list (S1 Appendix): Y (yes) = criterion met, N (no) = criterion not met, P (partial) = criterion partially met, and U (Unable to determine) = criterion unable to be determined. Generally scoring is as follows: Y = 1, N = 0, U = 0. However for item 5, Y = 2, P = 1, N = 0.

#### Participants

Five studies included participants only with TKA [30, 32, 37, 45, 47], 5 studies included participants with only THA [31, 33, 38, 40, 46], 1 study included participants with only UKA [43], and 6 studies included participants with either THA, TKA, or UKA [29, 34–36, 39, 50] (Table 3). Details of all included studies including demographic data, outcome measures, interventions in each group, and results are presented in Table 3. A total of 26,614 participants were included across all 17 studies with a mean age range of 50.3 to 72.3 years. The experimental group had a mean age range of 50.3 to 72.3 years, compared to 52.3 to 71.3 years in the control group. Among the studies that reported the distribution of male and female participants (15 studies), 27.1% were male and 72.9% were female (Table 3).

**Table 3:** Data extracted from the included studies.

Study	Participants Age, y ± SD, (range)	Interventions in Experimental Group	Interventions in Comparison / Control Group	Summary of Results
Chen A *et al* 2012, [29] USA, Prospective cohort study	Primary elective THA / TKA POD 1 PT (CG) *n* = 111 43 M, 61 F* 63.2 ± 11.0 POD 0 PT (EG) *n* = 25 10 M, 14 F* 58.0 ± 9.4	Hospitalized DOS, informed of ambulation before DC. POD 0: Patients are transferred to PACU, then inpatient care floor. Patients with TKA began CPM from 0°-90°. Patients are encouraged to get OOB and ambulate. If unable to ambulate, patient was moved from bed to chair. POD 1+: strengthening exercises initiated, progress exercises as appropriate. Exercises included isometric gluteal, quadriceps, hamstring, and hip adductor muscle sets. AAROM / AROM exercises included short-arc quadriceps ROM, long-arc quadriceps ROM, SLR, hip abduction, ankle pumps, and heel slides. Transfers, gait training, and ADLs also included.	Hospitalized DOS, informed of ambulation before DC. POD 0: Patients are transferred to PACU, then inpatient care floor. Patients with TKA began CPM from 0°-90°. Patients remained in bed or were moved from bed to chair, but did not ambulate. POD 1+: strengthening exercises initiated, progress exercises as appropriate. Exercises included isometric gluteal, quadriceps, hamstring, and hip adductor muscle sets. AAROM / AROM exercises included short-arc quadriceps ROM, long-arc quadriceps ROM, SLR, hip abduction, ankle pumps, and heel slides. Transfers, gait training, and ADLs also included.	*Outcome Measures*: *LOS* Statistically significant between group differences in mean LOS favoring EG.
Chen H *et al* 2012, [30] Taiwan, Retrospective study	TKA No rehabilitation (CoG) *n* = 5,533 1,497 M, 4,036 F 70.08 ± 8.1 After 2 weeks (CG) *n* = 1,570 397 M, 1,173 F 69.30 ± 8.5 Within 2 weeks (EG) *n* = 14,040 3,351 M, 10,689 F 69.56 ± 7.9	EG received inpatient or outpatient rehab services within 2 weeks after DC. Treatment protocol included: isometric exercise including ankle pumping, CPM, PROM including knee extension and flexion with heel slides (Range 0–90°), ambulation with walker, WB, and stationary cycling.	CoG did not receive rehab services after DC. CG received inpatient or outpatient rehab services more than 2 weeks after DC. Treatment protocol included: isometric exercise including ankle pumping, CPM, PROM including knee extension and flexion with heel slides (Range 0–90°), ambulation with walker, WB, and stationary cycling.	*Outcome Measures*: *Total medical expenses* Statistically significant between group differences in total medical expenses with CoG demonstrating the lowest overall costs and CG demonstrating the highest overall costs.
den Hertog *et al* 2012, [44] Germany, RCT	TKA Standard (CG) *n* = 73 20 M, 53 F 68.25 ±7.91 Fast-track (EG) *n* = 74 23 M, 51 F 66.58 ± 8.21	Joint Care^®^ fast-track rehabilitation implemented, received group therapy on DOS, stayed in three-bed hospital units, improved logistical organization involving case manager, provided with positive messages, informed that early discharge was scheduled for POD 6 as long as discharge criteria was met, competitive care implemented by comparing progress to fellow patients, DOS+: mobilized, 2 hours of standard intensive physiotherapy with focus on ADL, walking exercises, knee PROM flexion-extension up to 90-00-00°, lower limb muscle strengthening, respiratory training, group therapy. 18 day daily exercise program in rehabilitation center following DC from hospital.	Received standard perioperative care based on the individual’s subjective reports, stayed in three-bed hospital units, medication and discharge planning was discussed when the patient felt ready, not informed about intended LOS. DOS: intravenous fluid program POD 2+: first mobilization, 1 hour of physiotherapy exercises consisting of walking exercises, knee PROM flexion-extension up to 90-00-00°, lower limb muscle strengthening, respiratory training. 18 day daily exercise program in rehabilitation center following DC from hospital.	*Outcome Measures*: *LOS* Statistically significant between group differences in mean LOS favoring EG.
Gulotta *et al* 2011, [46] USA, Prospective cohort study	THA Traditional (CG) *n* = 134 80 M, 54, F 52.3 ± 8.5 Fast-track (EG) *n* = 149 98 M, 51 F 50.3 ± 8.9	Followed fast-track clinical pathway with a DC goal of 2 days, daily patient goals were outlined, surgery scheduled for first or second case of the day to allow for PT on DOS, educated about fast-track protocol, counseled about pain control and PT regimens, daily goals were outlined. DOS: ambulated x 3 with physical therapist, first time within 6 hours following surgery, progressed to WBAT with walker. POD 1: PT in the morning, attempt to progress to crutches or cane, PT in afternoon, progress to stairs if tolerated. POD 2: PT in the morning, progress to cane, work on stairs/transfers. D/C home if cleared by medical, surgical, and PT teams. Call home day after and 1 week after DC to screen for complications.	Followed the hospital’s traditional clinical pathway with a DC goal of 4 days. DOS: no ambulation POD 1: ambulated with PT No further information was provided.	*Outcome Measures*: *LOS* Statistically significant between group differences in mean LOS favoring EG.
Isaac *et al* 2005, [47] United Kingdom, Prospective cohort study	TKA Control (CG) *n* = 80 71.3 ± 8.1, (42–84) Accelerated (EG) *n* = 50 72.3 ± 9.9, (50–88)	Attended pre-assessment clinic, educated about rapid rehab and return home. DOS: mobilized using walker 4 hours post-op, SLR exercises, pillow under heel of operated leg to ensure full extension. POD 1: ROM to quadriceps and hamstrings, ambulation with walker. POD 2: ambulation with walker or walking stick. POD 3+: stair negotiation. Home visits 1, 2, 7 days post DC. Outpatient visit at 2, 6 weeks post DC.	Attended pre-assessment clinic. Rehab approach similar to intervention except, DOS: no PT. No home visits 1, 2, 7 days post DC.	*Outcome Measures*: *LOS* Statistically significant within group reduction in mean LOS for both the EG and CG, with a greater reduction in mean LOS favoring EG.
Juliano *et al* 2011, [31] USA, Retrospective descriptive study	Primary unilateral THA POD 1 (CG) *n* = 204 106 M, 98 F 60.4, (27–82) DOS (EG) *n* = 204 109 M, 95 F 60.2, (32–83)	3 days LOS clinical pathway used. DOS: treatment B/S, evaluation, dangle, stand, or ambulate as tolerated, B/S exercises, instructed on THA precautions. POD 1: treatment B/S, transfer training, progress ambulation distance as tolerated with walker, review exercises and precautions, high chair sitting, bathroom privileges. POD 2: treatment in PT gym, transfer training, attempt gait progression to cane or crutches, stair training, progress exercises. POD 3: treatment in PT gym, transfer training, progress gait, stairs, review HEP and ADL technique, DC if appropriate.	4 day LOS clinical pathway used. DOS: no PT intervention POD 1: treatment B/S, evaluation, dangle, stand, or ambulate as tolerated, B/S exercises, instruction on THA precautions. POD 2: treatment B/S, transfer training, progress ambulation distance as tolerated with walker, B/S exercises, review exercises and precautions, high chair sitting, bathroom privileges. POD 3: treatment in PT gym, transfer training, attempt gait progression to cane or crutches, stair training, progress exercises, review precautions, high chair sitting, bathroom privileges. POD 4: treatment in PT gym, transfer training, progress gait, stairs, review HEP and ADL technique, DC if appropriate.	*Outcome Measures*: *LOS* Statistically significant between group differences in mean LOS favoring EG.
Labraca *et al* 2011, [32] Spain, RCT	Primary TKA for OA Control (CG) *n* = 135 25 M, 110 F 66.36 ± 5.03 Intervention (EG) *n* = 138 37 M, 101 F 65.48 ± 4.83	POD 1: patient and family educated on rehab plan, PROM and AAROM knee flexion-extension from 0°-40°, isometric quadriceps and hamstring exercises with alternating 5-sec contract-relax, ankle flexion-extension for 10 minutes, active assisted anterior flexion of leg in extension, diaphragmatic breathing, education on posture. POD 2: same as day 1, in-bed sitting posture, transfer from bed to chair, standing, short-distance ambulation, management of AD, learning seated flexion-extension exercises, isotonic muscle work. POD 3: same as day 2, intensified exercises with AD, increased ambulation distance, ADLs. POD 4+: active-resisted quadriceps exercises, gait re-education, increased ambulation distance, stair negotiation with simulator, intensified muscle work, increased adaptation to ADLs	POD 1: no treatment, remained at rest in bed or chair POD 2: patient and family educated on rehab plan, PROM and AAROM knee flexion-extension from 0°-40°, isometric quadriceps and hamstring exercises with alternating 5-sec contract-relax, ankle flexion-extension for 10 minutes, active assisted anterior flexion of leg in extension, diaphragmatic breathing, education on posture. POD 3: same as day 1, in-bed sitting posture, transfer from bed to chair, standing, short-distance ambulation, management of AD, learning seated flexion-extension exercises, isotonic muscle work. POD 4: same as day 2, intensified exercises with AD, increased ambulation distance, ADLs. POD 5+: active-resisted quadriceps exercises, gait re-education, increased ambulation distance, stair negotiation with simulator, intensified muscle work, and increased adaptation to ADLs.	*Outcome Measures*: *LOS* Statistically significant between group differences in mean LOS favoring EG.
Larsen *et al* 2008, [33] Denmark, Quasi-experimental study	Primary elective THA Control (CG) *n* = 48 27 M, 23 F† 67 ± 9.8 Intervention (EG) *n* = 50 28 M, 27 F† 65 ± 9.6	Educated with 1 relative in groups about surgery and accelerated procedure at information day Friday before surgery, individual consults, hospitalized in new accelerated unit DOS, patient’s own clothes to be worn for LOS. DOS: mobilization. POD 1: goal of 4 hours OOB, training with PT and OT. POD 2+: goal 8+ hours mobilization. Outpatient follow-up 3 months post DC.	Hospitalized day before surgery, placed in general orthopedic ward, hospital clothes to be worn for LOS, educated individually day before surgery. POD 1: mobilized OOB, began training. POD 2+: mobilization increased to reach DC criteria, rehab adjusted to meet patient’s immediate state, care given to meet patient’s actual needs. Outpatient follow-up 3 months post DC.	*Outcome Measures*: *LOS*
Larsen *et al* 2009, [34] Denmark, Cost-effectiveness study based on RCT (Larsen, 2008)	Primary elective THA / TKA / UKA Standard (CG) *n* = 42 23 M, 19 F 66 ± 9.2 Accelerated (EG) *n* = 45 20 M, 25 F 64 ± 10.8	Educated in groups at outpatient clinic visit prior to hospitalization, hospitalized day of DOS, placed together with patients involved in study on separate part of ward, one nurse in charge of multidisciplinary team of nurses, OTs, and PTs, nutrition screening and focus on daily consumption of 1.5L of fluid, including two protein beverages. DOS: began mobilization and exercise. POD 1+: intensive mobilization of patients in teams following preset daily goals, 8 hours of mobilization daily.	Educated individually on day of admission, hospitalized day before surgery, placed randomly in general ward among other patients who were not part of study, various nurses in charge of care, various OTs and PTs responsible for mobilization, nutrition screening. POD 1: began mobilization and exercise. POD 2+: individual and gradual mobilization according to patient’s tolerance, 4 hours of mobilization daily.	*Outcome Measures*: *Total cost* Statistically significant between group differences in mean cost savings favoring the EG in patients with THA and TKA.
Larsen *et al* 2008, [35] Denmark, Prospective before-after trial	Primary elective THA / Primary elective TKA Control (CG) *n* = 105 53 M, 52 F 65 ± 11.0 Intervention (EG) *n* = 142 68 M, 74 F 65 ± 11.0	Educated with 1 relative in groups about procedure and plan for DC at information day week before surgery, individual consults, hospitalized in separate male and female beds in new accelerated unit DOS, patient’s own clothes to be worn for LOS. DOS: mobilization. POD 1: goal 4 hours OOB, training with PT and OT. POD 2+: goal 8+ hours mobilization. OOB activity (70% of mob time), gait training (15% of mob time), and exercises (15% of mob time). Exercises included hip and knee muscle strengthening, avoiding restricted motions; increased intensity, repetitions, and progression of acceleration as compared to CG.	Hospitalized day before surgery, placed in orthopedic ward, hospital clothes to be worn for LOS, educated about plan and procedure. POD 1: training in bed before lunch, mobilized OOB by PT after lunch. POD 2+: mobilized avg. 4 hours, mobilization increased to reach DC criteria, OOB activity (50% of mob time), gait training (25% of mob time), and exercises (25% of mob time). Exercises included hip and knee muscle strengthening, avoiding restricted motions.	*Outcome Measures*: *LOS* Statistically significant between group differences in mean LOS favoring EG.
Larsen *et al* 2008, [36] Denmark, RCT	THA / TKA / UKA Control (CG) *n* = 42 23 M, 19 F 66 ± 9.2 Intervention (EG) *n* = 45 20 M, 25 F 64 ± 10.8	Educated with 1 relative in groups about surgery and accelerated procedure at information day Friday before surgery, individual consults, hospitalized in new accelerated unit DOS, patient’s own clothes to be worn for LOS. DOS: mobilization. POD 1: goal of 4 hours OOB, training with PT and OT. POD 2+: goal 8+ hours mobilization. Outpatient follow-up 3 months post DC.	Hospitalized day before surgery, placed in general orthopedic ward, educated individually day before surgery, hospital clothes to be worn for LOS. POD 1: training in bed before lunch, mobilized OOB after lunch. POD 2+: mobilization increased to reach DC criteria, rehab adjusted to meet patient’s immediate state, care given to meet patient’s actual needs. Outpatient follow-up 3 months post DC.	*Outcome Measures*: *LOS* Statistically significant between group differences in mean LOS favoring EG.
Pua & Ong 2014, [37] Singapore, Retrospective cohort study	Primary elective unilateral TKA for OA Late ambulation on POD 2 (CG) *n* = 701 123 M, 578 F 66.8 ± 8.1 Early ambulation on POD 1 (EG) *n* = 803 183 M, 620 F 66.1 ± 7.6	Managed using coordinate clinical pathway. POD 1: began standard PT intervention including knee ROM, muscle strengthening exercises, began ambulation.	Managed using a coordinated clinical pathway. POD 1: began standard PT intervention including knee ROM, muscle strengthening. POD 2: began ambulation.	*Outcome Measures*: *LOS*, *Total hospitalization costs on DC* Statistically significant between group differences in mean LOS favoring EG. Statistically significant between group differences in mean costs favoring EG.
Raphael *et al* 2011, [50] Canada, Retrospective cohort study	THA / TKA Standard (CG) *n* = 100 47 M, 53 F 69 ± 8 Fast-track (EG) *n* = 100 52 M, 48 F 65 ± 9	Educated about fast-track program and expected plan for DC on POD 2, attended pre-surgical clinic several week prior to surgery. Following surgery patients were transferred to PACU and then SSU when they met PACU DC criteria. DOS: began physiotherapy in SSU 2–4 hours following surgery, 1–2 sessions of physiotherapy, bed transfers, sit to stand transfers, progressing to 5–10 minute ambulation with assistance from two staff members and AD, deep breathing, ankle pumps, static quadriceps, buttock exercises. Patients discharged to home or tertiary care facility when they met DC criteria. Contacted by nurse practitioner 2–3 days after DC to assess symptoms and recovery.	All surgery performed at tertiary care hospital, limited in preoperative education, no predetermined LOS plan, minimal DC planning prior to admission. DOS: no PT intervention POD 1: physiotherapy initiated if tolerated No further information was provided.	*Outcome Measures*: *LOS* Mean LOS was shorter in the EG than in the CG.
Reilly *et al* 2005, [45] UK, RCT	UKA Standard (CG) *n* = 20 63 Accelerated (EG) *n* = 21 63 24 M, 17 F total^‡^	Facilitated DC and DC support provided, goal of DC 24 hours following surgery. DOS: mobilized using walking frame two hours after surgery given the patient was alert and sufficiently pain free, progressed to ambulation using elbow crutches, stair negotiation, use of pain diary, Patients instructed on home use of pain diary, rehabilitation instructions, and potential problems. Patients educated to rest limb in extension, flex knee within limits of bandage, and use extension splint for ambulation for the first 5 days. Patients attended outpatient session with physiotherapist 5 days following DC for wound check and ROM assessment. Sutures removed, ROM assessed, and progression to one or two sticks occurred at appointment with physiotherapist 10–14 days following surgery. ROM assessed and observation by physiotherapist at 6 weeks post surgery.	Standard preparation for DC, urgency for deadlines not emphasized as it was with EG. Patient provided with pain diary and postoperative rehabilitation instructions. No further information was provided.	*Outcome Measures*: *LOS*, *total cost* Mean LOS was shorter for EG than CG. Average total cost was greater for CG than EG.
Robbins *et al* 2014, [38] USA, Retrospective cohort study	THA Control (CG) *n* = 400 188 M, 212 F Accelerated (EG) *n* = 190 99 M, 91 F 58.6, (31–87)	Patient and healthcare team education emphasized anticipated 24–48 hour LOS and DC to home, patients transferred from PACU to patient care unit by stretcher, unit staff received special education and instruction on post-op care of this patient cohort, mobilization and gait training implemented DOS for transfer from stretcher to hospital bed with walker or crutches, stand pivot transfer or slide transfer used for patients unable to begin gait training upon admission to hospital unit.	Patients transferred from PACU to patient care unit by hospital bed, mobilization initiated POD 1.	*Outcome Measures*: *LOS* Statistically significant between group difference in mean LOS favoring the EG.
Tayrose *et al* 2013, [39] USA, Prospective cohort study	THA / TKA Standard rehab (CG) *n* = 569 216 M, 353 F 64.3 Rapid rehab (EG) *n* = 331 125 M, 206 F 63.7	DOS: mobilized in recovery room, progress standard rehab protocol throughout LOS. Protocol includes progression of hang legs over side of bed, transfer to chair, ambulation, and climbing stairs.	POD 1: progress standard rehab protocol throughout LOS. Protocol includes progression of hang legs over side of bed, transfer to chair, ambulation, and climbing stairs.	*Outcome Measures*: *LOS* Statistically significant between group differences in mean LOS favoring EG.
Wellman *et al* 2011, [40] USA, Prospective cohort study	THA Control (CG) *n* = 209 Accelerated (EG) *n* = 218 97 M, 121 F 57.3, (23.5–79.9)	DOS: Patients are transferred from OR to PACU, then to hospital floor on stretcher, not hospital bed. Upon arrival, patients stand in hallway and walk to hospital bed with bilateral assistance, mobilized by PT or nursing staff. More senior or frail patients stand and pivot B/S instead of ambulation. Patients are encouraged to get up with PT or nursing staff one to several times daily and to walk to bathroom.	DOS: Patients are transferred from OR to PACU, then to hospital bed. Patients remain in bed to following morning.	*Outcome Measures*: *LOS* Mean LOS was shorter in the EG and resulted in faster DC to home.

Abbreviations: AAROM, active-assistive range of motion; AROM, active range of motion; ADL, activities of daily living; AD, assistive device; AVG, average; BOS, base of support; B/S, bed side CG, comparison group; CPM, continuous passive motion; CoG, control group; DOS, day of surgery; DC, discharge; EG, experimental group; F, females; HRQOL, health-related quality of life; HEP, home exercise program; LOS, length of stay; M, males; OT, occupational therapy; OR, operating room; OA, osteoarthritis; OOB, out of bed; PROM, passive range of motion; PT, physical therapy; PACU, post-anesthesia care unit; POD, post-operative day; RCT, randomized clinical trial; ROM, range of motion; SSU, short stay unit; SLR, straight leg raise; THA, total hip arthroplasty; TKA, total knee arthroplasty: TKR, total knee replacement; UKA, unicompartmental knee arthroplasty; WB, weight-bearing; WBAT, weight-bearing as tolerated

* The number of male and female participants is not reflective of the sample size (n = 111, n = 25) as n represents the total number of joints replaced.

^*†*^ The number of male and female participants is not reflective of the sample size (n = 48, n = 50) because in this study the authors reported total sample size after losses to follow-up were taken into account. The exact number of male and female drop-outs were not reported in the study.

^*‡*^ The number of male and female participants was not reported for each group; only the total ratio was provided.

#### Experimental group

Patients in the experimental groups received early rehabilitation following surgery. Early rehabilitation was defined as rehabilitation that commenced on either the day of surgery, or post-operative day one. This operational definition of early rehabilitation applied to 16 out of 17 studies. One study, however, had a slightly different methodological design in which three separate groups were analyzed: [30] an experimental group, a comparison group, and a control group. For this particular study, early rehabilitation is defined relative to the two other treatment groups and was initiated within the first two weeks following surgery. The authors would suggest that this is not the ideal timeframe for early initiation of rehabilitation, nevertheless, this article demonstrated differences in outcomes between groups.

#### Comparison / Control group

Patients in the comparison groups received standard or delayed rehabilitation following surgery. Standard rehabilitation was defined as rehabilitation that commenced on either post-operative day one, or post-operative day two. Delayed rehabilitation was defined as rehabilitation that commenced after two weeks of the surgery, which was only implemented in the Chen *et al* [30] study. This study also implemented a control group in which no formal rehabilitation was performed.

### Outcomes

The outcome measures reviewed in this manuscript included LOS (15 studies) [29, 31–33, 35–40, 43, 45–47, 50] and cost (4 studies) associated with treatment [30, 34, 37, 43] (Table 3). Data from the included articles, including data in the experimental group, data in the comparison/control group, as well as between group differences are presented in Table 4.

**Table 4:** Results of included studies.

Study	Experimental Group	Comparison / Control Group	Between group difference
***Randomized Clinical Trials***			
den Hertog *et al* 2012 [44]	Mean LOS, days 6.75 ± 2.92*	Mean LOS, days 13.20 ± 1.63*	Shorter mean LOS for EG than CG (p < .0001) **Calculated difference: 6.45**
Labraca *et al* 2011 [32]	Mean LOS ± SD, days 6.37 ± 1.16	Mean LOS ± SD, days 8.46 ± 2.36	Shorter mean LOS for EG than CG (p < .001) **Calculated difference: 2.09**
Larsen *et al* 2008 [36]	Unadjusted mean LOS ± SD 4.9 ± 2.4	Unadjusted mean LOS ± SD 7.8 ± 2.1	Adjusted mean difference in LOS (95% CI; p-value) 3.1 (2.3–4.0; p < .001) **Calculated difference: 2.9**
Reilly *et al* 2005 [45]	Mean LOS ± SD, days 1.5 ± 0.7	Mean LOS ± SD, days 4.3 ± 1.3	Mean LOS was shorter for EG than CG. **Calculated difference: 2.8**
	Average total cost, £ 3,391	Average total cost, £ 4,634	Average saving per patient in EG was **£1243**, approximately 27% saving on the total average cost per patient in CG.
***Cost-effectiveness Study based on a Randomized Clinical Trial***			
Larsen *et al* 2009 [34]	Average total cost ± SD, DKK 71,344 ± 39,958	Average total cost ± SD, DKK 90,227 ± 47,475	Uni-variate crude analysis of mean difference in incremental average total cost, DKK (95% CI; p-value) **–18,880** (–1899 to –38,152; p = .036)
***Quasi-experimental Study***			
Larsen *et al* 2008 [33]	Mean LOS ± SD, days 4.2 ± 1.8	Mean LOS ± SD, days 7.3 ± 1.8	Mean LOS was shorter for EG than CG. **Calculated difference: 3.1**
***Prospective Cohort Study***			
Chen A *et al* 2012 [29]	Mean LOS ± SD, days 2.81 ± 0.77	Mean LOS ± SD, days 3.79 ± 1.74	Shorter mean LOS for EG than CG (p = .019) **Calculated difference: 0.98**
Gulotta *et al* 2011 [46]	Mean LOS ± SD, days 2.6 ± 0.9	Mean LOS ± SD, days 4.1 ± 1.5	Shorter mean LOS for EG than CG (p < .0001) **Calculated difference: 1.5**
Isaac *et al* 2005 [47]	Mean LOS ± SD, days 3.6 ± 1.0	Mean LOS ± SD, days 6.6 ± 2.6	Greater reduction in mean LOS favoring EG (p < .001) **Calculated difference: 3.0**
Larsen *et al* 2008 [35]	Mean LOS ± SD, days 4.3 ± 1.8	Mean LOS ± SD, days 8.8 ± 3.0	Crude adjusted mean difference in LOS, days (95% CI; p-value) 4.4 (3.8–5.0; p < .001) **Calculated difference: 4.5**
Tayrose *et al* 2013 [39]	Mean LOS, days 3.85	Mean LOS, days 4.39	Shorter mean LOS for EG than CG (p < .001) **Calculated difference: 0.54**
Wellman *et al* 2011 [40]	Mean LOS ± SD, days 1.65 ± 0.89	Mean LOS ± SD, days 3.54 ± 1.05	Mean LOS was shorter for EG than CG. **Calculated difference: 1.89**
***Retrospective Cohort Study***			
Chen H *et al* 2012 [30]	Mean total medical expenses ± SD, NTD EG 4,603.42 ± 9,551.60	Mean total medical expenses ± SD, NTD CoG 141.66 ± 3,350 CG 6,913.98 ± 10,661.53	CoG had lowest total medical expenses, while CG had highest total medical expenses (p < .001) **Calculated difference: 2,310.56**
Juliano *et al* 2011 [31]	Mean LOS ± SD, days 3.27 ± 0.85	Mean LOS ± SD, days 3.48 ± 0.88	Shorter mean LOS for EG than CG (p = .014) **Calculated difference: 0.21**
Pua & Ong 2014 [37]	Adjusted LOS, days 4.07	Adjusted LOS, days 4.51	Adjusted mean difference in LOS (95% CI; p-value) **–0.44** (–0.29 to 0.60; p < .001)
	Adjusted Total Hospitalization Costs on DC, S$ 11,215	Adjusted Total Hospitalization Costs on DC, S$ 11,600	Adjusted mean difference in costs (95% CI; p-value) **–385** (–112 to –672; p < .001)
Raphael *et al* 2011 [50]	Adjusted Mean LOS, days† 1.96 (95% CI 1.71–2.21)	Adjusted Mean LOS, days† 4.83 (95% CI 4.58–5.08)	Adjusted mean difference in LOS (95% CI) **–2.88** (–2.5 to –3.25)
Robbins *et al* 2014 [38]	Mean LOS, days 2.06 (Range 1–9)	Mean LOS, days 3.38 (Range 1–23)	Shorter mean LOS for EG than CG (p < .05) **Calculated difference: 1.32**

Abbreviations: CG, comparison group; CI, confidence interval; DKK, Danish kroner; DC, discharge; EG, experimental group; HRQOL, health-related quality of life; LOS, length of stay; £, pounds; ROM, range of motion; S$, Singapore dollars; SD, standard deviation; VAS, visual analog scale

*Standard deviations were obtained by contacting the author.

^†^Mean LOS data was converted from hours to days for consistency.

#### Length of stay

**Fig 2:**
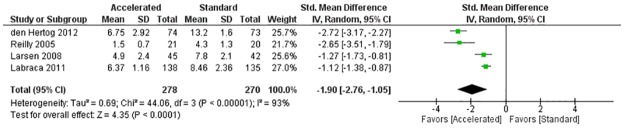
Length of stay for RCTs meta-analysis. Abbreviations: df, degrees of freedom; IV, inverse variance; RCT, randomized clinical trial; Std, standardized.

**Fig 3:**
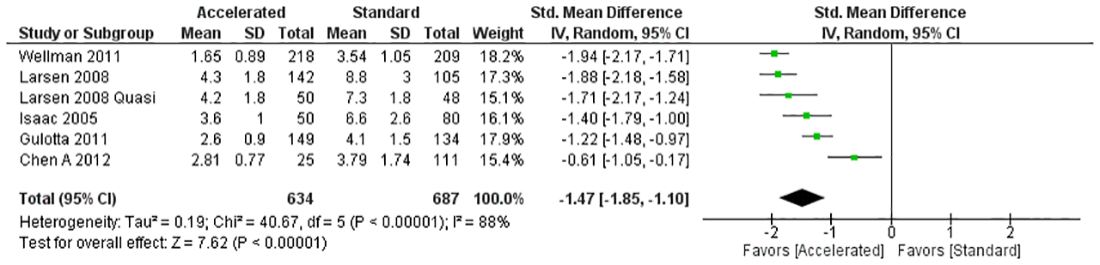
Length of stay for prospective and quasi-experimental studies meta-analysis. Abbreviations: df, degrees of freedom; IV, inverse variance; Std, standardized.

All 15 studies that measured LOS as an outcome variable demonstrated a shorter hospital stay in the group receiving early physical therapy, with 12 of the studies demonstrating a statistically significant reduction in LOS following THA, TKA, or UKA (Table 4) [29, 31, 35–40, 45–47, 50]. While the Larsen *et al* [33], Reilly *et al* [43], and Wellman *et al* [40] studies did not calculate inferential statistics, a shorter mean LOS favoring the experimental group was reported (Table 4). The results of the meta-analysis that included the four RCTs (n = 548) demonstrated a large effect size favoring the early rehabilitation group (standardized mean difference = -1.90; 95% CI -2.76 to -1.05; I^2^ = 93%) (Fig 2). The results of the meta-analysis that included 6 studies (5 prospective studies and 1 quasi-experimental study, n = 1,321) demonstrated a large effect size favoring the early rehabilitation group (standardized mean difference = -1.47; 95% CI -1.85 to -1.10; I^2^ = 88%) (Fig 3). Both meta-analyses however, demonstrated a large I^2^ value indicating substantial heterogeneity among studies.

#### Costs

Of the four studies that measured cost as an outcome variable [30, 34, 37, 43], two, Larsen *et al* [34] and Pua and Ong [37], demonstrated a statistically significant between group mean cost savings favoring the experimental group that received early post-operative physical therapy. The third study by Chen *et al* [30], was unique to this systematic review because it implemented a third group, in which participants did not receive physical therapy. The findings of this study were similar to those of Larsen *et al* [34] and Pua and Ong [37] that demonstrated a statistically significant between group mean cost savings favoring the experimental group (Table 4). It was not appropriate to conduct a meta-analysis on costs due to differing methodological designs in the studies assessing costs. Overall, three studies [30, 34, 43] assessed total medical expenses following joint arthroplasty, while one study, Pua and Ong [37], only assessed hospitalization costs at discharge.

## Discussion

The results of this systematic review demonstrated an overall reduction in LOS (15 studies) [29, 31–33, 35–40, 43, 45–47, 50] and cost (4 studies) [30, 34, 37, 43] in the group that received early rehabilitation following joint arthroplasty, without any increase in adverse reactions compared to participants in the standard rehabilitation group. This is consistent with other research in which early physical therapy has resulted in a shorter LOS following proximal hip fractures and hemiarthroplasty [51–53]. In addition, research has reported that early physical therapy can decrease adverse reactions such as venous thromboembolism following THA and TKA [54–56]. While the meta-analyses demonstrated an overall decrease in LOS favoring the experimental group with large effect sizes, the heterogeneity of the included studies were also high (Figs 2 and 3). In addition, lack of detail provided about the interventions in the different groups makes it difficult to assess and replicate the treatment factors that led to early discharge from the acute care setting.

Findings from the current systematic review exhibit both similarities and differences with the previous systematic review on early mobilization following hip or knee joint arthroplasty. Guerra *et al* [41] conducted a systematic review and meta-analysis, that focused only on RCTs and demonstrated a moderate to large effect size favoring early mobilization. In contrast, the authors of the current review chose to allow a variety of study designs, including prospective and retrospective cohort studies to achieve a broader overview of the literature. Thus, the current review includes several cohort studies that have large sample sizes, and two RCTs considered by Guerra *et al* [41] (Dowsey *et al* [57] and Whitney *et al* [58]) were excluded because they did not meet current inclusion criteria. While prospective and retrospective cohort studies represent a lower quality of evidence, they have the potential to bridge the gap between research and clinical practice, thereby increasing generalizability of findings. Moreover, the strict internal rigors necessary for RCTs may not be logistically possible in many clinical settings and thus not reflect the manner in which the intervention is likely to be applied in practice. Despite the lesser quality of evidence considered, the authors believe that the included cohort studies add important clinical information that must be combined with RCTs to provide a comprehensive summary of optimal treatment interventions.

Clinicians may question the safety of these early mobilization algorithms and the potential for an increase in adverse reactions. Of the 17 studies identified in this systematic review, 11 reported adverse reactions [30, 35–38, 40, 43, 45–47, 50], examples of which can be found in Table 5. Three studies, Larsen *et al* [35], Pua and Ong [37], and Gulotta *et al* [46] reported no statistically significant differences in the number of adverse reactions between groups. Raphael *et al* [50] reported no statistically significant differences in number of emergency department visits or re-admissions to the hospital within 30 days following surgery between groups. Chen *et al* [30] reported a statistically significant difference (p < .0001) in the number of adverse reactions between groups, however, the percentage of participants who developed prosthetic infection was lower in the group that received rehabilitation in the first 2 weeks. Moreover, the group that received rehabilitation within the first two weeks had a lower rate of deep vein thrombosis [30]. The remaining six studies [36, 38, 40, 43, 45] did not calculate between group differences for the number of adverse reactions.

**Table 5:** Adverse effects associated with included studies.

Study	Experimental Group Adverse Effects	Comparison / Control Group Adverse Effects
Chen H *et al* 2012 [30]	Prosthetic infection, results not indicated DVT, results not indicated	Prosthetic infection, results not indicated DVT, results not indicated
den Hertog *et al* 2012 [44]	Deep infection Stiffness Urinary tract infection Subluxation of the patella Tibial fissure	Humerus fracture Stiffness Urinary tract infection Subluxation of the patella Tibial fissure
Gulotta *et al* 2011 [46]	Dislocation, resulting in re-admission treated with a closed reduction in emergency department Periprosthetic femoral fracture, resulting in re-admission treated with a femoral revision Early aseptic loosening, resulting in re-admission treated with acetabular cup revision Significant anemia, resulting in re-admission treated with blood transfusion Gastrointestinal bleed POD 1, resulting transfer to medical ICU Pulmonary embolus POD 1, leading to atrial fibrillation	Dislocation, resulting in re-admission treated with open reduction and revision of prosthesis
Isaac *et al* 2005 [47]	Delayed recovery in renal function, resulting in hospital stay extended 7 days Suspected DVT, resulting in re-admission to hospital. Subsequent duplex ultrasound scanning demonstrated no DVT Failure to cope at home, with associated leg and ankle pain, resulting in re-admission to hospital	
Larsen *et al* 2008 [35]	One major perioperative complication related to THA implant—not indicated, resulting in no effect on LOS Respiratory arrest after pneumonia, resulting in death Re-admission to hospital, adverse effects not indicated	Re-admission to hospital, adverse effects not indicated Respiratory arrest after pneumonia, resulting in death
Larsen *et al* 2008* [36]	Swelling and pain in knee, resulting in re-admission, LOS 11 days Hip dislocation, resulting in re-admission, LOS 1 day	PE, resulting in death day after surgery Wound Infection, resulting in re-admission to hospital, revision surgery, additional LOS 15 days
Pua & Ong 2014 [37]	Re-admission to hospital, adverse effects not indicated	Re-admission to hospital, adverse effects not indicated
Raphael *et al* 2011 [50]	Intra-operative electrocardiogram changes, resulting in increased LOS 3–4 days. Subsequent cardiology consultation and dobutamine stress echocardiogram demonstrated no coronary ischemia. Chest pain, resulting in increased LOS 3–4 days. Subsequent cardiology consultation and dobutamine stress echocardiogram demonstrated no coronary ischemia. Post-operative hematoma and infection, resulting in re-admission Emergency department visits, adverse effects not indicated Re-admission to hospital, adverse effects not indicated	Inadequate pain control, resulting in re-admission Emergency department visits, adverse effects not indicated Re-admission to hospital, adverse effects not indicated
Reilly *et al* 2005 [45]	Bleed following removal of drain Poor ROM (50 degrees of flexion), resulting in re-admission to hospital treated with MUA Superficial wound infection Episode of low blood pressure Suspected DVT. Subsequent testing demonstrated no DVT.	Deep wound infection, resulting in re-admission to hospital treated with outpatient antibiotics Suspected DVT. Subsequent testing demonstrated no DVT.
Robbins *et al* 2014 [38]	Right thigh hematoma, resulting in re-admission to hospital, LOS 7 days secondary to muscle spasms limiting functional mobility; pain; and symptomatic anemia	Hip pain, resulting in re-admission to hospital Hip infection, resulting in admission to hospital’s ambulatory care unit, intravenous antibiotics administered Significant leg length discrepancy detected in PACU, returned to OR same DOS for THA revision Other complications: tachycardia, hypoxia, postoperative anemia, atelectasis, lower extremity swelling, atrial fibrillation, pneumonia, confusion, ileus, respiratory arrest, bradycardia, elevated INR, and lower extremity hematoma
Wellman *et al* 2011 [40]	I&D for acutely increasing pain, resulting in re-admission 8 months post-op	

Abbreviations: DVT, deep vein thrombosis; DOS, day of surgery; I&D, incision and drainage; ICU, intensive care unit; INR, international normalized ratio; POD, post-operative day; LOS, length of stay; MAU, manipulation under anesthesia; OR, operating room; PACU, post-anesthesia care unit; PE, pulmonary embolism; ROM, range of motion; THA, total hip arthroplasty

*Adverse events were reported in the follow-up cost effectiveness study (Larsen et al, 2009), but are not reported here because the study was based on the Larsen et al 2008 RCT and are, therefore, the same.

This review suggests that early initiation of physical therapy can be carried out without increased risks to patients when properly designed treatment paradigms are implemented. In practice, quality assurance measures should be in place to ensure that earlier discharge from the inpatient hospital setting does not lead to a higher re-admission rate following THA and TKA. Before widespread implementation of treatment paradigms that promote earlier initiation of physical therapy and quicker discharge from the hospital setting, the authors would recommend that future studies be conducted with less heterogeneity of treatment interventions and stronger methodological quality focusing on principles of both internal and external validity.

With the exception of the previous discussion on adverse reactions, no other long-term outcomes or quality of life measures were considered across the included studies, preventing the authors of this systematic review from making any further conclusions. In order to assess the long-term outcomes of early initiation of rehabilitation following joint arthroplasty, larger prospective cohort studies focusing on functional outcomes and quality of life measures with a one to two year follow-up are required.

With the costs of healthcare continuing to rise, it is necessary that all treatment interventions be assessed for cost-effectiveness. In this systematic review, 4 studies assessed cost-effectiveness as a primary outcome variable [30, 34, 37, 43]. Two studies, Larsen *et al* [34] and Pua and Ong [37] showed a statistically significant reduction in mean cost favoring the experimental group, which received earlier physical therapy. These results are similar to a recent systematic review published by Ojha *et al* [28].

One included study conducted by Chen *et al* [30] had a third group, in which no physical therapy was provided (Table 3). This study reported that the group that did not receive rehabilitation demonstrated the lowest total medical expenses compared with the group that received physical therapy within 2 weeks and the group that received physical therapy after 2 weeks. However, Chen *et al* [30] cautioned that while the group which did not receive physical therapy treatment demonstrated lower costs overall, patients in this group were more likely to experience a higher incidence of prosthetic infection (odds ratio 1.29, p = .0409) and deep vein thrombosis (odds ratio 1.51, p = .0099) compared to those in the group that initiated physical therapy within a 2 week time frame [30]. While it is impossible to identify a definitive reason for the increase in adverse reactions, one plausible theory is that increased blood pooling in the lower extremities associated with longer periods of inactivity after surgery may increase the risk of deep vein thrombosis. The authors would encourage clinicians and health policy makers to view these results in context and avoid making premature decisions that no physical therapy is the best option following joint replacement surgery. Since the studies included in this review focused on LOS and costs, there were no validated outcome measures implemented to assess treatment effectiveness. However, the authors suggest that the clinical effectiveness of these early initiation programs can be manifested in both decreased costs to the healthcare system, as well as an improved quality of life, less overall complications, and faster discharge from the hospital setting.

Although the authors’ original intention was to perform meta-analysis on costs as well as length of stay, differences in sample size, methodological design, reported data, and forms of currency hindered further statistical analysis. In accordance with the Center for Medicare and Medicaid Services’ [59] recently implemented initiative, total medical costs are bundled together over a 90-day period, including skilled nursing facility (SNF), rehabilitation, visiting nurse services, emergency department visits, and office visits. While this serves as the standardized definition of total medical costs in the US, other countries may have different reimbursement systems. Since the four included studies that discussed cost were from countries other than the US, it is necessary to consider various operational definitions of costs within this systematic review. Nonetheless, early initiation of physical therapy demonstrated a consistent trend in cost savings across studies.

To our knowledge, this systematic review provided the most comprehensive summary to date assessing the role of early initiation of physical therapy on LOS and costs following joint arthroplasty. The results of this systematic review suggest that patients in the group that received early physical therapy intervention had shorter LOS, lower medical cost, and experienced no greater risk of adverse events. This review is a timely contribution to the literature because recent healthcare reform has renewed providers’ interest in shortening the LOS after hip and knee replacement procedures. A policy established on April 1, 2016 mandates hospitals located in 67 geographic areas, defined by metropolitan statistical areas, to participate in the Comprehensive Care for Joint Replacement (CCJR) model [59]. In this model, participant hospitals are financially accountable for the quality and cost of care of patients receiving joint replacement procedures in an episode between hospital admission and 90 days post-discharge. Participant hospitals have the financial incentives to discharge patients as early as possible when clinically appropriate. Traditionally, Medicare patients receiving joint replacement procedures had to stay in the hospital for at least 3 days to qualify for Medicare covered SNF stay. As part of the CCJR payment model, patients could have the 3-day qualifying hospital stay waived as long as they are discharged to a SNF that has an overall 3-star rating or better under the Centers for Medicare and Medicaid Services 5-Star Quality Rating System.

While timing of physical therapy initiation may be one critical factor, another important consideration may be the overall plan of care. Among the included studies, treatment protocols varied in the pre-surgical, rehabilitation, and post-surgical management strategies. Prior to surgery, the following conditions varied among studies: type of education provided before surgery, timing of hospital admission, location of residence within the hospital, and healthcare practitioners involved in the care. Rehabilitation and post-surgical follow-up varied in the number of hours and treatment sessions per day, types of interventions performed, and whether or not home care services were provided (Table 3). While variability is inevitable between treatment protocols following joint arthroplasty, all components of rehabilitation should be addressed within a biopsychosocial management approach that considers the multi-faceted aspects of patient care.

### Study limitations

Several limitations exist in this review. Thirteen of 17 studies were non-randomized clinical trials, suggesting that confounding variables may have had an influence on the results. However, there was no indication across studies that natural selection of participants’ ability to comply with the protocol of early rehabilitation played a role in group allocation. The results of this study can only be generalized to patients in the inpatient setting who specifically had THA, TKA, or UKA with similar interventions. In addition, the lack of homogeneity of the data prevented the authors from performing a meta-analysis on total medical costs. Finally, neither the dosage nor type of intervention was standardized across studies, which may have had an influence on post-operative outcomes. Future systematic reviews may consider selecting inclusion criteria that address the specifics of the rehabilitation protocol including dosage and frequency of physical therapy in the acute care setting.

## Conclusions

This review suggests that early initiation of physical therapy following THA, TKA, or UKA is associated with a shorter LOS and lower overall cost. Furthermore, the results presented in this review show no evidence of increased number of adverse effects when physical therapy is initiated early following joint replacement surgery.

## Supporting information

S1 Table PRISMA 2009 checklist. (DOC)

S2 Table Modified Downs and Black checklist. (DOCX)
